# Characterization and expression profiling of ATP-binding cassette transporter genes in the diamondback moth, *Plutella xylostella* (L.)

**DOI:** 10.1186/s12864-016-3096-1

**Published:** 2016-09-27

**Authors:** Weiping Qi, Xiaoli Ma, Weiyi He, Wei Chen, Mingmin Zou, Geoff M. Gurr, Liette Vasseur, Minsheng You

**Affiliations:** 1 Institute of Applied Ecology and Research Centre for Biodiversity and Eco-Safety, Fujian Agriculture and Forestry University, Fuzhou, 350002 China; 2 Fujian-Taiwan Joint Innovation Centre for Ecological Control of Crop Pests, Fujian Agriculture and Forestry University, Fuzhou, 350002 China; 3 Key Laboratory of Integrated Pest Management of Fujian and Taiwan, China Ministry of Agriculture, Fuzhou, 350002 China; 4 Graham Centre, Charles Sturt University, Orange, NSW 2800 Australia; 5 Department of Biological Sciences, Brock University, 1812 Sir Isaac Brock Way, St. Catharines, ON L2S 3A1 Canada

**Keywords:** ABC transporter, Phylogenetic analysis, Transcriptome analysis, Insecticide resistance, Detoxification

## Abstract

**Background:**

ATP-binding cassette (ABC) transporters are one of the major transmembrane protein families found in all organisms and play important roles in transporting a variety of compounds across intra and extra cellular membranes. In some species, ABC transporters may be involved in the detoxification of substances such as insecticides. The diamondback moth, *Plutella xylostella* (L.), a destructive pest of cruciferous crops worldwide, is an important species to study as it is resistant to many types of insecticides as well as biological control *Bacillus thuringiensis* toxins.

**Results:**

A total of 82 ABC genes were identified from our published *P. xylostella* genome, and grouped into eight subfamilies (ABCA-H) based on phylogenetic analysis. Genes of subfamilies ABCA, ABCC and ABCH were found to be expanded in *P. xylostella* compared with those in *Bombyx mori*, *Manduca sexta*, *Heliconius melpomene*, *Danaus plexippus*, *Drosophila melanogaster*, *Tetranychus urticae* and *Homo sapiens*. Phylogenetic analysis indicated that many of the ABC transporters in *P. xylostella* are orthologous to the well-studied ABC transporter genes in the seven other species. Transcriptome- and qRT-PCR-based analysis elucidated physiological effects of ABC gene expressions of *P. xylostella* which were developmental stage- and tissue-specific as well as being affected by whether or not the insects were from an insecticide-resistant strain. Two ABCC and one ABCA genes were preferentially expressed in midgut of the 4th-instar larvae of a susceptible strain (Fuzhou-S) suggesting their potential roles in metabolizing plant defensive chemicals. Most of the highly expressed genes in insecticide-resistant strains were also predominantly expressed in the tissues of Malpighian tubules and midgut.

**Conclusions:**

This is the most comprehensive study on identification, characterization and expression profiling of ABC transporter genes in *P. xylostella* to date. The diversified features and expression patterns of this gene family may be associated with the evolutionary capacity of this species to develop resistance to a wide range of insecticides and biological toxins. Our findings provide a solid foundation for future functional studies on specific ABC transporter genes in *P. xylostella*, and for further understanding of their physiological roles and regulatory pathways in insecticide resistance.

**Electronic supplementary material:**

The online version of this article (doi:10.1186/s12864-016-3096-1) contains supplementary material, which is available to authorized users.

## Background

ATP-binding cassette (ABC) transporters constitute one of the largest transmembrane protein families, which is widespread across all species. The first ABC transporter was found in prokaryotes, and the first cloned and characterized human ABC transporter member was ABCB1, which confers multidrug resistance (MDR) to cancer cells, preventing the accumulation of chemotherapeutic drugs [1]. According to their functions, the ABC proteins can be divided into three categories: importers, exporters and non-transport proteins [2]. Importers are only found in prokaryotes. In eukaryotes, ABC transporters are exporters and involved in the excretion of drugs, and endogenous and exogenous toxins. The third class of ABC proteins are apparently not related to molecule transport, but rather acting as ion channels, regulators of ion channels and receptors, and in some cases involved in DNA repair, ribosome assembly and translation [3].

ABC transporters are generally composed of four core domains with two being nucleotide-binding domains (NBDs) that bind and hydrolyze ATP and two transmembrane domains (TMDs) mediating translocation of the respective substrate [4]. Reflecting the diversity of substrates handled by ABC transporters, TMDs are much more diverse than NBDs, whose sequences are highly conserved in order to perform their roles as ATP hydrolyzing enzymes [5]. Half-transporters consist of one NBD and one TMD and need to form homo- or heterodimers to be functional [6]. The mode of action of ABC transporters is called “ATP-switch” transport cycle [7, 8]. The ABC family is classified into eight subfamilies, annotated A to H according to their sequence similarity and domain conservation [3]. The H subfamily appears to be present in all insects, mites, the slime mould *Dictyostelium*, and zebrafish, but is absent in genomes of plants, worms, yeasts, and mammalian species [9, 10]. In humans, many ABC proteins have been characterized with special functions and mutations of ABC genes can cause or contribute to a series of genetic disorders [11].

ABC transporters have recently been documented in insects as a family of detoxification-involved proteins [12], complementing the activity of another three classes of major metabolic enzymes: cytochrome P450 monooxygenases (P450s), glutathione S-transferases (GSTs) and carboxylesterases (COEs). Detoxification of toxins occurs in three phases with the first phase being associated predominantly with P450s [13]. In phase II, GSTs are dominant and known to be linked to resistance development to most classes of insecticides [14]. A series of transporters, including members of ABC transporters, are involved in phase III, which aims at the elimination of products generated during phases I and II [15]. Some ABC members of subfamilies B, C and G are involved in resistance to xenobiotics including insecticides [12]. Epis et al. [16] report that combining the insecticide permethrin with the ABC transporter inhibitor leads to greater *Anopheles stephensi* mortality than when using permethrin alone, demonstrating the importance of ABC transporters in insecticide resistance. Besides their detoxification roles, RNAi-mediated knockdown of some ABC genes in *Tribolium castaneum* results in a series of abnormal developmental phenotypes, such as growth arrest, eye pigmentation defects, abnormal cuticle formation, egg-laying and egg-hatching defects, and mortality [17].

The insect pest *Plutella xylostella* is a cosmopolitan Lepidoptera that almost exclusively feeds on cruciferous plants [18]. Due to its short life cycle and capacity to rapidly develop insecticide resistance, *P. xylostella* is difficult to control [19, 20]. The species is the first to be reported resistant to dichlorodiphenyltrichloroethane (DDT) in the 1950s [21] and *Bacillus thuringiensis* (Bt) toxins in the 1990s [22]. Bt resistance of *P. xylostella* is associated with *ABCC2* alone [23] or in combination with *ABCC3* [24] or *ABCG1* [25]. In addition, the silencing of an *ABCH1* gene results in the death of larvae and pupae [26]. Expression of ABC genes is found to be more frequently up-regulated than that of GSTs, COEs or P450s in insecticide-resistant larvae of *P. xylostella*, suggesting a potential detoxification role of ABC transporters [27].

In this study, based on the ABC transporter genes (*PxABCs*) previously identified from the *P. xylostella* genome [27], we further characterized the gene structure and motifs, and performed phylogenetic analysis using *P. xylostella*, *Bombyx mori*, *Manduca sexta*, *Heliconius melpomene*, *Danaus plexippus*, *Drosophila melanogaster*, *Tetranychus urticae*, and *Homo sapiens* to further understand the evolutionary relationships among the eight subfamilies identified in this study. In addition, we carried out transcriptome- and qRT-PCR-based expression profiling of the ABC transporter genes in different developmental stages, tissues, and insecticide-susceptible and resistant strains of *P. xylostella*.

## Results and discussion

### Identification and grouping of the PxABCs

Based on our previous work on annotation of *PxABCs* in the *P. xylostella* genome [27], we identified 82 ABC transporter genes (Table 1 and Additional file 1) and 19 ABC fragments (Additional file 2). The 19 ABC fragments had homology to ABC transporters of other insects, but lacked the highly conserved NBDs of canonical ABC proteins [4]. *P. xylostella* ABC transporter genes were grouped into the eight subfamilies (A-H) (Additional file 3). The number of genes in each subfamily greatly varied, ranging from one gene in ABCE to 21 in ABCC (Table 2). The ABCC subfamily was further divided into two groups with one group highly similar to the ABCB subfamily, which was also found in the other Lepidoptera, *B. mori* [28].

**Table 1:** Description of subfamily-based ABC transporter genes identified in the *P. xylostella* genome

Subfamily	Gene ID	Protein (aa)	Scaffold	Position	No. exons	RNA-seq^a^	qRT-PCR^b^
ABCA1	Px001174	969	scaffold_1135	304…19217	19	+	−
ABCA2	Px004981	1266	scaffold_188	295012…323041	28	+	−
ABCA3	Px004982	1186	scaffold_188	301796…321736	26	+	−
ABCA4	Px008069	1933	scaffold_288	24893…72425	42	+	−
ABCA5	Px008254	1086	scaffold_295	204319…225237	19	+	−
ABCA6	Px008255	739	scaffold_295	226197…247340	17	+	−
ABCA7	Px008256	1569	scaffold_295	250906…269981	29	+	−
ABCA8	Px009697a	233	scaffold_346	259984…265745	6	+	−
ABCA9	Px009697b	1296	scaffold_346	286234…303713	24	+	−
ABCA10	Px009783	1852	scaffold_35	644704…664586	37	+	−
ABCA11	Px013614	3796	scaffold_568	132102…192807	72	+	+
ABCA12	Px013659	786	scaffold_570	19302…30379	18	+	+
ABCA13	Px016911	1195	scaffold_856	62…22277	35	+	+
ABCA14	Px016912	2714	scaffold_856	24266…52676	49	+	−
ABCA15	Px017838	4008	scaffold_97	202450…263565	73	+	−
ABCB1	Px000163	1219	scaffold_10	1427959…1440157	22	+	−
ABCB2	Px002636	658	scaffold_14	2273580…2292376	18	+	−
ABCB3	Px002801	959	scaffold_142	423653…432357	17	+	−
ABCB4	Px004522	124	scaffold_175	1104754…1106729	3	+	−
ABCB5	Px005591	1257	scaffold_200	68221…86053	24	+	−
ABCB6	Px006391	862	scaffold_225	442449…454413	17	+	−
ABCB7	Px007221	1568	scaffold_254	255160…283670	30	+	−
ABCB8	Px007992	823	scaffold_284	306641…321481	18	+	−
ABCB9	Px008679	1218	scaffold_306	348764…361734	23	+	−
ABCB10	Px009649	912	scaffold_344	75019…96464	18	+	−
ABCB11	Px012211	1279	scaffold_479	179910…193122	23	+	−
ABCB12	Px013177	329	scaffold_53	1202314…1212357	7	+	−
ABCB13	Px013728	1308	scaffold_58	462696…478688	26	+	−
ABCB14	Px013729	1699	scaffold_58	487550…504266	31	+	−
ABCC1	Px001274	548	scaffold_1153	4862…13438	12	+	−
ABCC2	Px002416	1327	scaffold_49	2357…26234	2	+	+
ABCC3	Px002415	1406	scaffold_137	488447..513162	26	+	−
ABCC4	Px002418	912	scaffold_137	552867…576380	16	+	+
ABCC5	Px002419	471	scaffold_137	576715…581467	9	+	+
ABCC6	Px002696	1881	scaffold_140	577784…598125	27	+	−
ABCC7	Px002784	1069	scaffold_142	99127…105692	15	+	−
ABCC8	Px004042	1098	scaffold_164	402013…423822	22	+	−
ABCC9	Px005403	1274	scaffold_199	854564…879035	24	+	−
ABCC10	Px005931	143	scaffold_21	2289257…2290685	3	+	−
ABCC11	Px005932	451	scaffold_21	2289194…2309332	14	+	−
ABCC12	Px006710	1397	scaffold_236	4405…26723	24	+	−
ABCC13	Px008999	1818	scaffold_316	108977…138269	29	+	+
ABCC14	Px009134a	662	scaffold_321	178075…188823	14	+	−
ABCC15	Px009134b	861	scaffold_321	194897…231476	27	+	−
ABCC16	Px009834	1333	scaffold_352	169022…191602	24	+	+
ABCC17	Px009835	1262	scaffold_352	192939…220411	24	+	+
ABCC18	Px012780	891	scaffold_51	11388…27660	15	+	−
ABCC19	Px014427	806	scaffold_627	73391…97213	19	+	−
ABCC20	Px015447	710	scaffold_712	47919…65879	12	+	−
ABCC21	Px015888	211	scaffold_749	69505…71718	3	+	−
ABCD1	Px007715	700	scaffold_274	451324…468854	18	+	+
ABCD2	Px010231	765	scaffold_372	135778…153573	16	+	+
ABCD3	Px010704	619	scaffold_395	100478…120592	9	+	−
ABCE1	Px007660	267	scaffold_271	5031…10235	11	+	−
ABCF1	Px001241	901	scaffold_115	254134…267193	18	+	−
ABCF2	Px002158	621	scaffold_132	19709…32348	11	+	+
ABCF3	Px006766	657	scaffold_239	104170…121670	14	+	+
ABCG1	Px000087	575	scaffold_1	1687310…1700888	8	+	−
ABCG2	Px001524	646	scaffold_120	391766…407227	11	+	−
ABCG3	Px002116	141	scaffold_131	33576…47509	2	+	−
ABCG4	Px002117	490	scaffold_131	50160…54319	9	+	−
ABCG5	Px004725	583	scaffold_180	233059…240664	11	+	+
ABCG6	Px005467	518	scaffold_2	1210934…1215177	9	+	−
ABCG7	Px007185	587	scaffold_252	315812…338592	12	+	−
ABCG8	Px007949	1239	scaffold_282	234990…275031	22	+	−
ABCG9	Px007950	614	scaffold_282	282382…298666	12	+	−
ABCG10	Px008370	787	scaffold_3	1084031…1102351	15	+	−
ABCG11	Px008371	689	scaffold_3	1107251…1133659	14	+	+
ABCG12	Px012058	763	scaffold_47	714983…763358	12	+	−
ABCG13	Px016406	691	scaffold_8	151167…158192	12	+	−
ABCG14	Px016407	653	scaffold_8	190187…201753	13	+	−
ABCG15	Px016675	639	scaffold_82	554151…570599	10	+	−
ABCG16	Px016677	641	scaffold_82	593974…605475	10	+	+
ABCG17	Px016679	603	scaffold_82	593448…622103	14	+	−
ABCG18	Px017344	499	scaffold_909	2090…8847	10	+	−
ABCG19	Px017858	736	scaffold_97	716767…726144	7	+	−
ABCH1	Px003594	778	scaffold_158	256543…275266	16	+	−
ABCH2	Px004510	693	scaffold_175	841069…867340	12	+	−
ABCH3	Px005110	821	scaffold_19	1346939…1358906	13	+	−
ABCH4	Px005111	813	scaffold_19	1363123…1425269	15	+	−
ABCH5	Px014955	781	scaffold_677	19741…43381	14	+	+
ABCH6	Px014956	899	scaffold_677	45879…80000	17	+	−

^a^ “+” represents the gene could be found in the *P. xylostella* transcriptome

^b^ “+” represents the gene was validated by qRT-PCR, and “-” represents the gene was not validated

**Table 2:** Numerical distribution of subfamilies (A - H) based on ABC transporter genes of different species

Species	A	B	C	D	E	F	G	H	Total	Reference
*Homo sapiens*	13	11	12	4	1	3	5	0	48	[3]
*Saccharomyces cerevisiae*	0	4	6	2	2	6	10	0	30	[31]
*Drosophila melanogaster*	11	8	14	2	1	3	15	3	57	[3]
*Tribolium castaneum*	10	6	35	2	1	3	13	3	73	[17]
*Apis mellifera*	3	5	9	2	1	3	15	3	41	[28]
*Daphnia pulex*	4	7	7	3	1	4	23	15	64	[33]
*Bombyx mori*	6	8	15	2	1	3	13	3	51	[28]
*Tetranychus urticae*	9	4	39	2	1	3	23	22	103	[10]
*Plutella xylostella*	15	14	21	3	1	3	19	6	82	[26] and the present study
*Manduca sexta*	7	10	9	2	1	3	16	3	51	The present study
*Danaus plexippus*	8	16	12	3	1	3	16	3	62	The present study
*Heliconius melpomene*	10	11	15	2	1	3	17	3	62	The present study

### Characterization of the PxABCs and their motifs

The 82 *PxABCs* were dispersed on 59 scaffolds, 40 of which were found being individually located on different scaffolds. The remaining *PxABCs* were clustered on 19 scaffolds with each containing two or three genes, suggesting tandem duplication of these genes. The length of most predicted ABC transporters ranged from 124 to 2,714 amino acids (aa), with two exceptionally long genes containing 3,796 and 4,008 aa. The corresponding exon numbers ranged from 2 to 73 (Table 1), showing high structural complexity (Additional file 3).

**Fig. 1:**
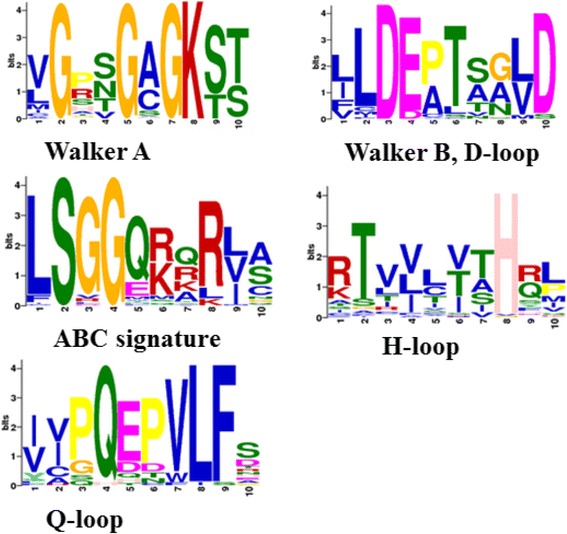
Illustration of six conserved motifs identified from the *P. xylostella* ABC transporters. The overall height of each column is proportional to the information content of all of the amino acids at that position, and within each of the columns the conservation of each residue is visualized as the relative height of symbols representing amino acids. Walker A, GxxGxGKST; Walker B, LLDEPT; D-loop, LD; ABC signature, LSGGQ; H-loop, xHx; Q-loop: xQx

The NBDs of ABC transporters generally contain seven highly conserved, but not invariant, motifs including Walker A, Walker B, ABC signature, A-loop, Q-loop, D-loop and H-loop [8]. The Walker motifs A and B show that the ATP binding sites [29] and the “signature sequence” (also called C-loop or LSGGQ motif) are exclusive to ABC transporters, contributing to the formation of a composite catalytic site [5]. Additional motifs such as the A-loop, Q-loop, D-loop and H-loop (also called the switch loop) only possess a single conserved aa residue [5], which are key amino acids in the catalytic cycle. We identified in *P. xylostella* six of these seven conserved motifs, with A-loop being absent (Fig. 1). However, the nature of such structural invariance in NBDs among arthropods remains poorly understood [30].

### Subfamily-based comparison of ABC transporters

#### ABCA

**Fig. 2:**
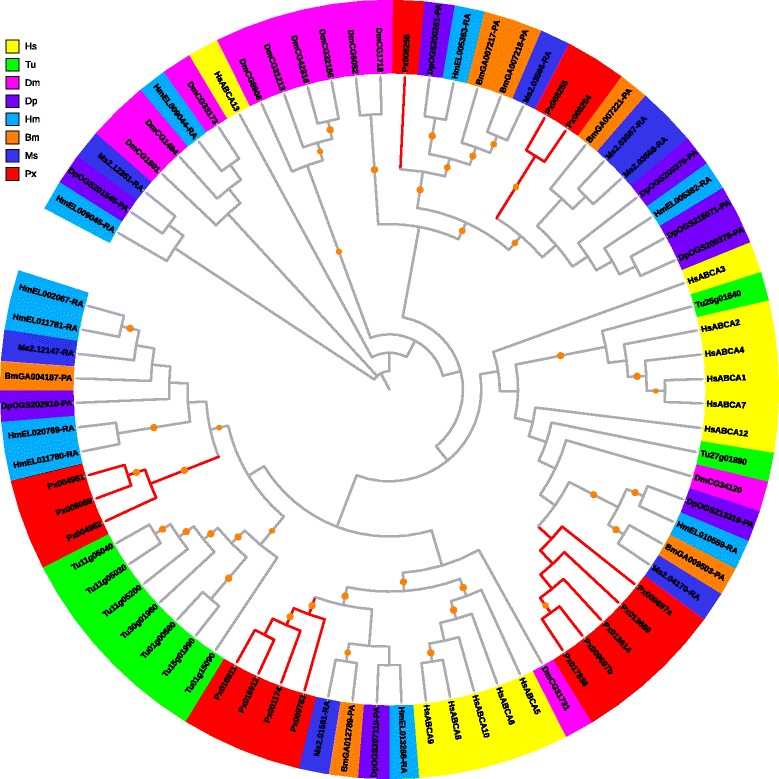
Phylogenetic analysis of ABCA transporters of eight species. Full-length ABCA proteins among eight species were aligned using MUSCLE, and subsequently to generate a phylogenetic tree using a maximum likelihood analysis with 1000 replications. Species are differentially coded with Hs for *H. sapiens*, Tu for *T. urticae*, Dm for *D. melanogaster*, Dp for *D. plexippus*, Hm for *H. melpomene*, Bm for *B. mori*, Ms for *M. sexta* and Px for *P. xylostella.* The branches with bootstrap support > 80 % were dotted in orange, and the ones harboring PxABCs were in red. Protein sequences used for phylogenetic analysis are provided in the Additional file 4

In *P. xylostella*, 15 ABCA genes were identified, including one 3-gene cluster on scaffold 295, two 2-gene clusters on scaffolds 188 and 856, and eight genes that were individually located on different scaffolds. Apparently, gene tandem duplication resulted in high protein diversity of ABCA. ABCA subfamily harbored the two longest ABC transporter genes, *Px013614* (3,796 aa) and *Px017838* (4,008 aa), which were similar in length to *B. mori* [28] and *T. urticae* [10]. In *Saccharomyces cerevisiae* (yeast), no ABCA member has been identified [31] (Table 2). ABCA transporters in human are characterized as full-transporters [3], however, we found their structures are variable in arthropods. The ABCA transporters of *T. urticae* and *D. melanogaster* were all full-transporters, while the moths and butterflies contained both full- and half-transporters (Additional file 4). The *P. xylostella* ABCA subfamily comprised of nine full- and six half-transporters. Phylogenetic analysis of the ABCA subfamily revealed a Lepidoptera-specific clade with a distinct expansion in *P. xylostella*, which was orthologous to a human-specific clade (Fig. 2). A same orthologous relationship was also observed between another Lepidoptera-specific clade and a mite-specific clade (Fig. 2).

ABCA transporters in human are involved in lipid transport and metabolism [3]. For example, the ABCA1 protein is mutated in a recessive disorder (Tangier disease) characterized by a defect in biogenesis of high density lipoprotein (HDL) [32]. It is reported that *HsABCA4* is linked to the Stargardt disease because of its role in retinal integrity, *HsABCA3* is involved in lung surfactant production, and *HsABCA12* participates in keratinization processes in the skin [11]. However, the roles of ABCA transporter genes in arthropods are currently unclear. They may share a function related to lipid trafficking processes based on the high conservation of their structure. RNAi-mediated knockdown of *TcABCA-9A* or *TcABCA-9B* results in ~30 % mortality of *T. castaneum* [17], implying their significant roles in insects.

#### ABCB

**Fig. 3:**
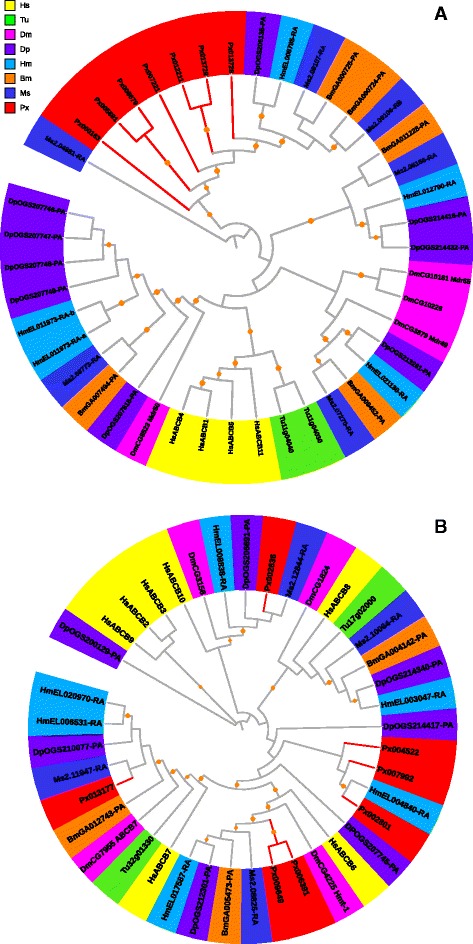
Phylogenetic analysis of full- (**a**) and half- (**b**) transporters in the ABCB subfamily of eight species. See the legend of Fig. 2 for performance and presentation details. Protein sequences used for phylogenetic analysis are provided in the Additional files 5 (full) and 6 (half)

ABCB subfamily contains both full- and half-transporters [33]. In the *P. xylostella* genome, we identified seven full- and seven half-transporters. The phylogenetic analysis of full-transporters showed that *H. sapiens* and *T. urticae* were located in a separate clade from insects (Fig. 3a). The phylogenetic tree of most ABCB half-transporters showed obvious orthologs and high bootstrap values among the eight species (Fig. 3b), indicating that they were orthologous and evolutionary divergent. Our results suggest that full-transporters may have evolved through lineage-specific duplication, while half-transporters may be evolutionarily conserved in metazoan species [10, 28, 33].

In human, the full-transporter ABCB1 plays important roles in cancer development by contributing to MDR [1]. Expression of the ABCB1 gene of *P. xylostella*, *PxPgp1*, was found being up-regulated in the strain subjected to insecticide abamectin [34]. In *Heliothis virescens*, increased expression of the human *Pgp* orthologs is associated with resistance to thiodicarb [35]. Mutations in bile salt export pump gene (BSEP, *HsABCB11*) and multidrug-resistant gene 3 (MDR3, *HsABCB4*) in human can result in progressive familial intrahepatic cholestasis [36]. Based on the phylogenetic tree (Fig. 3a), HsABCB4 and HsABCB11, both being full-transporters, seemed to be functioning in a human-specific manner. Human ABCB6, 7, 8 and 10 are mitochondrial half-transporters involved in iron metabolism and transport of Fe/S protein precursors [37]. Their orthologs in arthropods are clear (Fig. 3b), implying the similar roles as in humans [30]. However, the human-specific ABCB half-transporters (Fig. 3b), ABCB2, 3 and 9, are involved in antigen processing [37].

#### ABCC

**Fig. 4:**
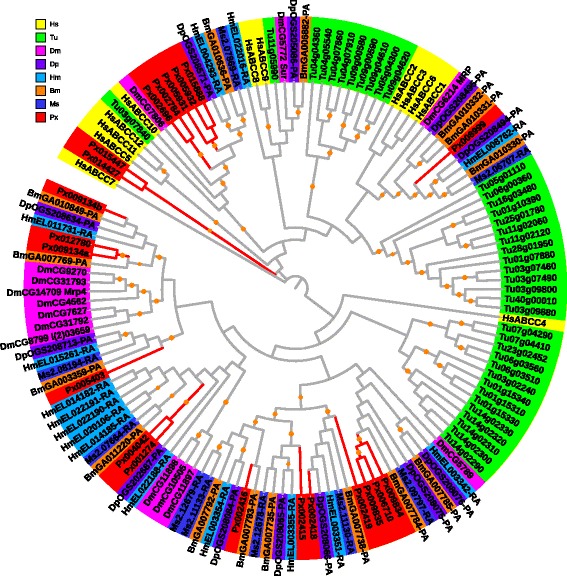
Phylogenetic analysis of ABCC transporters of eight species. See the legend of Fig. 2 for performance and presentation details. Protein sequences used for phylogenetic analysis are provided in the Additional file 7

In *P. xylostella*, ABCC transporters formed the largest subfamily with 21 members (Table 1). There were one 3-gene cluster on scaffold 137, two 2-gene clusters on scaffolds 21 and 321, and 14 genes that were individually located on different scaffolds, suggesting tandem duplication of those clustered genes. Human ABCC transporters are all full-transporters, but in insects both full- and half-transporters can be found [3, 15, 28]. It showed similar structural characteristics to what we have found in ABCA subfamily of the eight species. The *P. xylostella* ABCC subfamily consisted of eight full- and 13 half-transporters. The phylogenetic tree of ABCC transporters showed that lineage-specific members were significantly duplicated in *T. urticae*, while this kind of duplication was inconspicuous in other species, suggesting varying evolutionary divergence of ABCC subfamily among the studied species (Fig. 4).

ABCC transporters are regarded as multidrug-resistance associated proteins (MRPs) due to their ability to extrude drugs with broad specificity. In *D. melanogaster* adults, MRPs may play a role in secretion of methotrexate by the Malpighian tubules [38], which is a drug used for treating cancers and auto-immune disorders [39]. Labbe et al. propose that ABCC transporters can be involved in xenobiotic efflux of moths [12]. Similar to ABCB4, ABCC2 is also involved in bile acids, phospholipids, and bilirubin export and related to progressive familial intrahepatic cholestasis [36]. ABCC7 (cystic fibrosis transmembrane conductance regulator, CFTR) is an important ABC transporter involved in ion transport in human, and those who have mutations on functional CFTR may suffer diseases [40, 41]. However, no orthologs of human ABCC7 were found in *P. xylostella* and the other arthropod species.

Although mutations in ABCC2 have been implicated in Bt resistance in *H. virescens, P. xylostella*, *Trichoplusia ni,* and *B. mori* [23, 42–44], species-specific patterns of cross-resistance to Bt toxins are varied. This suggests that different mutations in ABCC2 may have occurred influencing the types of Bt resistance in a toxin binding site-dependent manner [45]. Recently, ABCC3 ortholog in *P. xylostella* (Px008999) has been reported to also be associated with Bt resistance in insects [24], while its ortholog in human (ABCC3) functions as a marker for MDR in non-small cell lung cancer [46].

ABCC transporters also have cell-surface receptor activity, such as sulfonylurea receptor (SUR) [47]. Among the eight species studied, *P. xylostella* and *M. sexta* had no ortholog of SUR (Fig. 4). In arthropods, SUR has been proposed as the direct target site for benzoylphenylureas (BPUs), a group of chitin synthesis inhibitors [48]. The insecticidal activity of BPUs in *P. xylostella* [49] provides a clue for further exploring its SUR gene. Recent research however suggests that SUR may not be the only receptor for chitin synthesis as shown in *D. melanogaster* embryos [50]. Knocking down SUR ortholog in *T. castaneum* has no effect on chitin synthesis [17].

#### ABCD

**Fig. 5:**
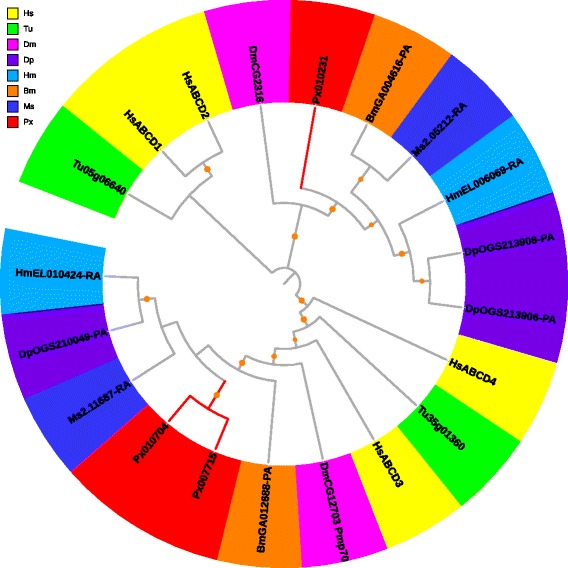
Phylogenetic analysis of ABCD transporters of eight species. See the legend of Fig. 2 for performance and presentation details. Protein sequences used for phylogenetic analysis are provided in the Additional file 8

The ABCD subfamily solely consists of half-transporters with the topology TMD–NBD, except for some plant representatives [47]. All three ABCD members in *P. xylostella* were half-transporters. ABCD subfamily was present in all studied taxa and comprised of two to five members (Table 2). The ABCDs showed four distinct groups with *HsABCD4* standing in its own branch and the three *PxABCDs* being clustered into two Lepidoptera-specific clades (Fig. 5). Their high orthologous relationships indicate that they are evolutionarily conserved in metazoan species.

ABCD transporters are peroxisomal membrane-located and are involved in importing fatty acids and/or fatty acyl-CoAs into peroxisome with specific metabolic and developmental functions in various organisms [51]. However, the role of ABCD transporters in arthropods is still unclear [30].

#### ABCE and ABCF

**Fig. 6:**
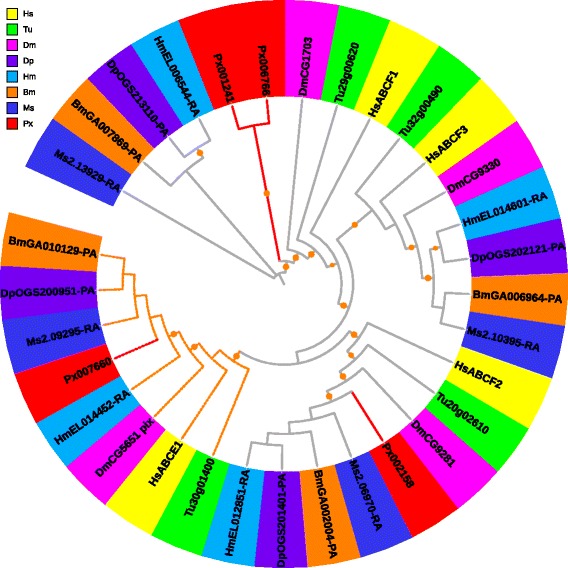
Phylogenetic analysis of ABCE and ABCF transporters of eight species. See the legend of Fig. 2 for performance and presentation details. The branches that harbor ABCE genes were presented in dotted line. Protein sequences used for phylogenetic analysis are provided in the Additional files 9 (ABCE) and 10 (ABCF)

ABCE proteins showed a high similarity (>50 % of aa identity) among the studied species (Fig. 6). The numbers of ABCE and ABCF were highly conserved, with most eukaryotes (including *P. xylostella*) having one ABCE and three ABCF genes (Table 2). Phylogenetic analysis showed that they had clear orthologous relationships (Fig. 6).

The structure of ABCE and ABCF are quite distinct from other ABC transporters due to their lack of TMDs and only containing a pair of linked NBDs [52]. Therefore, they are not related to transport. Initially, ABCE was identified as an RNase L inhibitor in human [53]. In human and yeast, ABCE proteins contribute in translation initiation [54]. ABCF is similar to ABCE and relates to ribosome biogenesis and translational control. RNAi-mediated knockdown of members in the ABCE and ABCF subfamilies in the flour beetle *T. castaneum* results in 100 % mortality in penultimate larvae, showing fundamental roles of ABCE and ABCF in biological processes of the insects [15]. In *P. xylostella*, the ABCE gene (*Px007660*) exhibited the highest expression level compared to other *PxABC*s based on the *P. xylostella* genome [27] and transcriptome [55]. The same result is also reported in *B. mori* [28].

#### ABCG

In *P. xylostella*, we found a total of 19 ABCG transporters, with 18 of them being half-transporters and only one full-transporter (Px007949). Gene duplication appeared to have occurred several times among ABCG genes of *P. xylostella*, which was evidenced by the location of gene paralogs on scaffolds 3, 8, 82, 131 and 282 (Table 1). Most ABCG transporters are half-transporters and they need to form homo- or hetero-dimers to perform the transport function [30].

**Fig. 7:**
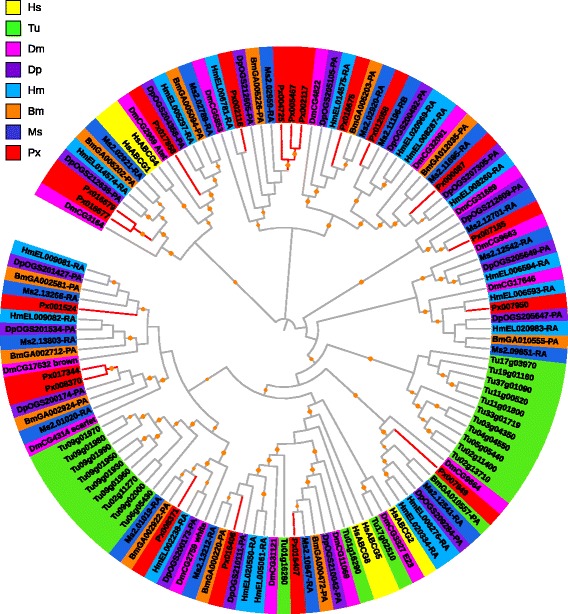
Phylogenetic analysis of ABCG transporters of eight species. See the legend of Fig. 2 for performance and presentation details. Protein sequences used for phylogenetic analysis are provided in the Additional file 11

In the phylogenetic tree of ABCG, all the orthologs of *Dmwhite, Dmbrown* and *Dmscarlet* were identified in Lepidoptera (Fig. 7). They are the most well-known ABCG genes in *D. melanogaster*, and encode for proteins to transport guanine or tryptophan precursors of the red and brown eye color pigments influencing the development of compound and simple eyes [56, 57]. These orthologs are also present in other insects, such as *Anopheles gambia*e [58], *Bactrocera dorsalis* [59] and *Ceratitis capitata* [60], suggesting that white, brown and scarlet are highly conserved protein transporters for eye color pigments in insects. Although several *P. xylostella* ABCG proteins have similar sequences with the white, brown and scarlet proteins in *D. melanogaster*, it is unclear whether they are involved in eye color pigments. It has been reported that *Pxwhite* is associated with Bt resistance in *P. xylostella* [25]. In *A. stephensi*, analysis shows that the ortholog of *Dmscarlet*, *AnstABCG4*, exhibits a ten-fold increased expression when treated with permethrin after 48 h compared to untreated control [16].

In human, ABCG proteins are involved in lipid transport across membranes. The most intensively studied ABCG in human is ABCG2 (breast cancer resistance protein, BCRP), which acts as a MRP transporting anticancer drugs and a series of substrates [61–63]. In our study, no human ABCG2 orthologs were identified in Lepidoptera (Fig. 7), which were identified in *D. melanogaster* (E23) and *T. urticae. D. melanogaster* E23 (*DmCG3327*) is a 20-hydroxyecdysone (20E) primary response ABC transporter, which can suppress 20E-mediated gene activation [64]. In *B. mori*, five ABCG genes including *BmABC005226, BmABC005203, BmABC005202, BmABC010555* and *BmABC010557*, are 20E responsive genes although they are not orthologous to *E23* [28]. The orthologous relationships of *HsABCG5* and *HsABCG8* with the other species showed their evolutionarily conserved function (Fig. 7), the translated proteins of which form a functional heterodimer that is coordinately regulated by cholesterol [3].

#### ABCH

**Fig. 8:**
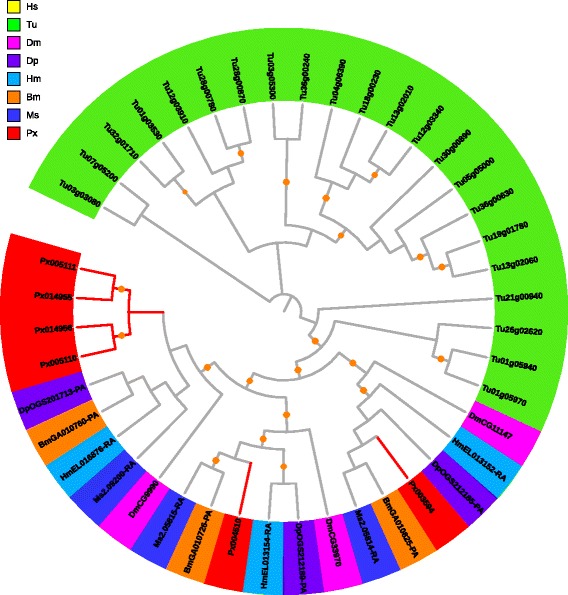
Phylogenetic analysis of ABCH transporters of seven arthropod species. See the legend of Fig. 2 for performance and presentation details. Protein sequences used for phylogenetic analysis are provided in the Additional file 12

We identified 6 ABCH genes in *P. xylostella* and they were all half-transporters. One of them (*Px004510*) tended to share high sequence similarity with ABCF subfamily (Additional file 3). ABCH subfamily was first identified with the sequencing of *D. melanogaster* genome [3]. The structure of ABCH proteins is most closely related to subfamily ABCG. A previous study using five insect species with available genomes suggests that insect ABCHs may have originated from a common ancestral copy [28]. Our phylogenetic analysis further supports this hypothesis (Fig. 8). Outside the Insecta, however, the *T. urticae* ABCHs seem not to conform to this rule (Fig. 8).

Down-regulation of an ABCH1 gene can cause high mortality of larvae and pupae of *P. xylostella* [26]. Similarly, RNAi-mediated knockdown of *TcABCH-9C* results in desiccation and 100 % mortality in *T. castaneum* [17], and knockdown of ABCH gene *CG9990* in *D. melanogaste*r is lethal [65, 66]. It may be inferred that ABCH is a newly evolved subfamily with specific functions in the development of certain species, but this needs to be investigated.

### Stage- and tissue-specific expression of the *PxABCs*

**Fig. 9:**
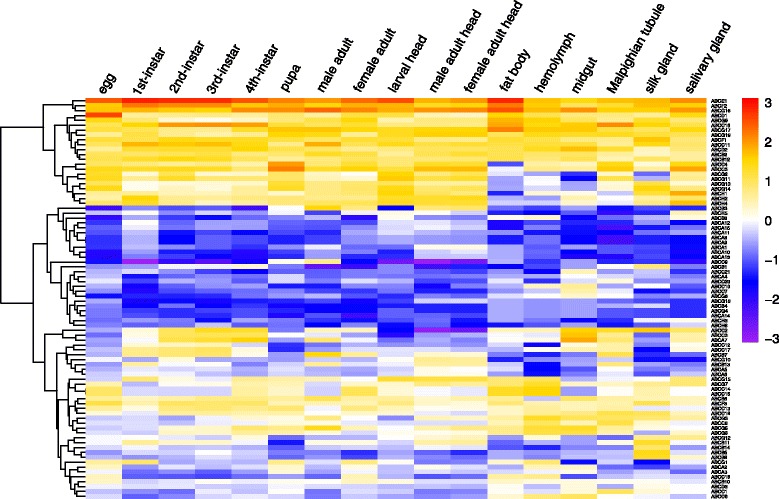
Expression patterns of the ABC genes in multiple developmental stages and tissues of *P. xylostella* based on RPKM values. The relative expression levels are illustrated by seven scaled colors and corresponding log_2_ RPKM values (Additional file 13) ranging from −3.00 to +3.00. Tissue-specific expression profiling was performed using multiple tissues (if not specified) of the 4th larvae as shown on the top of the figure. The colors vary from bright red showing up-regulated expression to bright purple for down-regulated genes

In order to develop an understanding of the physiological functions of ABC transporters in *P. xylostella*, the expression of 82 *PxABCs* was profiled at different developmental stages and with different tissues of the susceptible strain (Fuzhou-S, SS) (Fig. 9 and Additional file 13), based on the *P. xylostella* genome and transcriptome datasets [27, 55]. The RPKM values of the 82 ABC genes were clustered into three separate clades. The first clade contained 22 ABC genes with higher expression levels in various tissues and developmental stages, including two ABCDs, one ABCE and two ABCFs, which showed particularly high expression levels. This indicated that while ABCD, ABCE and ABCF were numerically small subfamilies (Table 2), they might be of some biological importance, though currently unclear. Within the second clade, there were 25 ABC genes exhibiting low expressions with most RPKM values being > 1.

The third clade contained 35 ABC genes with variable expression patterns. We found that *Px002415* (*ABCC3*), *Px002416* (*ABCC2*) and *Px008256* (*ABCA7*) were relatively highly expressed in midgut of 4th instar and all larval stages compared with the other tissues and stages. Previous studies have shown that ABCC2 proteins play important physiological roles in resistance to Bt toxins [23, 42, 43]. Since midgut is an important organ involved with food digestion and detoxification, and larva is the main feeding stage of *P. xylostella*, we suspect that these genes may also play important roles in metabolizing plant secondary compounds. Many ABC genes were preferentially expressed in Malpighian tubule and most of them belonged to ABCB and ABCC subfamilies (Fig. 9). This suggests that ABC transporter genes of these subfamilies may also be involved in xenobiotic detoxification because Malpighian tubules make up an important excretory organ for transporting wastes [15].

### Strain-specific expression profiles of the *PxABCs*

**Fig. 10:**
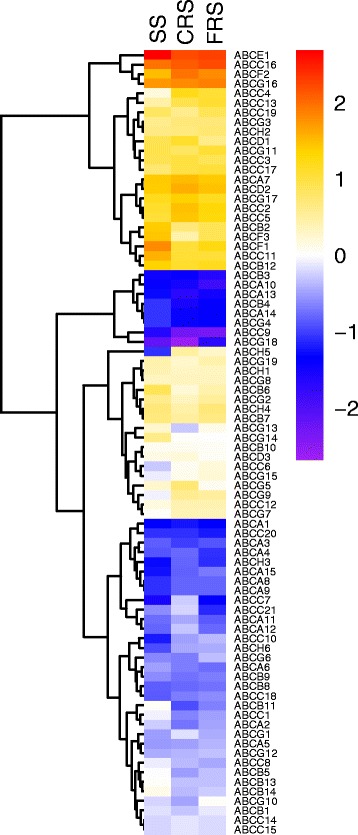
Strain-specific expression patterns of the ABC genes in the 3rd instar larvae of *P. xylostella* based on RPKM values. The relative expression levels are illustrated by seven scaled colors and corresponding log_2_ RPKM values (Additional file 14) ranging from −2.00 to +2.00. SS: susceptible strain; CRS: chlorpyrifos-resistant strains; FRS: fipronil-resistant strain

In order to understand the potential function of the *P. xylostella* ABC proteins in insecticide resistance, expressions of the *P. xylostella* ABC genes were profiled for larvae of two insecticide-resistant strains (fipronil (FRS) and chlorpyrifos (CRS)) (Fig. 10 and Additional file 14), based on the genome and transcriptome data and compared with the susceptible strain (SS) [27, 55]. The majority of the *P. xylostella* ABC genes were up-regulated in FRS and/or CRS, with a few genes being down-regulated. The ABCE gene (*Px007660*) showed the highest expression level in all three strains, suggesting its fundamental role in various physiological processes of the cell. At least, five ABCC genes including *Px002418* (*ABCC4*), *Px008999* (*ABCC13*), *Px009835* (*ABCC17*), *Px002416* (*ABCC2*), and *Px002419* (*ABCC5*) were up-regulated in FRS and CRS. Among them, ABCC genes are proposed to be involved in insecticide resistance [12], and ABCC2 is linked to Bt resistance [23].

In our case, one of the ABCH genes (*Px014955*) was mostly up-regulated in FRS and CRS, and weakly expressed in SS. Four genes including *Px002784* (*ABCC7*)*, Px013614* (*ABCA11*)*, Px013659* (*ABCA12*) and *Px015888* (*ABCC21*) showed up-regulated expression in CRS, but not in FRS, suggesting that the expression of these genes could be induced by specific insecticides. There were eight *PxABCs* exhibiting low expression (RPKM value < 1) in all three strains. Three of the *PxABC* genes (*ABCA14*, *ABCB4* and *ABCG4*) had no differential expression in the three strains, suggesting that they might not be related to the resistance of chlorpyrifos and fipronil. *Px006766* (*ABCF3*) and *Px013659* (*ABCA12*) were down-regulated in FRS and CRS, while *Px013614* (*ABCA11*)*, Px008371* (*ABCG11*) and *Px004725* (*ABCG5*) exhibited up-regulated expression in both insecticide-resistant strains. This may indicate that not all ABC transporter genes are responsible for xenobiotics excretion, and a strictly transcriptional regulation of *ABCs* in response to external challenges is required for balancing transmembrane substrate transport.

### qRT-PCR validation of expressions

Eighteen ABC transporter genes with up- or down-regulated expressions in the two insecticide-resistant strains (FRS and CRS) of *P. xylostella* were selected for experimental validation using qRT-PCR. Quantitative expression of the *PxABCs* at different developmental stages of the susceptible strain (Additional file 15) and two insecticide-resistant strains (Additional file 16) overall confirmed the stage- and strain-specific expression patterns as profiled using the *P. xylostella* genome and transcriptome datasets (Figs. 9 and 10). The results provide significant clues for further studies on functions of some specific *PxABCs* in host plant adaptation and insecticide resistance development.

## Conclusions

Our work presents the most comprehensive study to date on identification, characterization and expression profiling of ABC transporters in the genome of *P. xylostella*, integrating evolutionary and physiological aspects. A comparison of ABC transporters from seven arthropod species and human provides an overview of this vital gene family. The variances in genomic features and expression patterns of the genes reflect evolutionary and functional diversification of the ABC transporters. Some genes are preferentially expressed in larvae, midgut, and Malpighian tubules, which may be involved in detoxification of plant defense chemicals. Moreover, some of the ABC transporter genes are up-regulated in two insecticide-resistant strains compared to the susceptible strain, suggesting their involvement in detoxification of the chemical insecticides through transportation. Our work offers a solid foundation for further research on gene-specific functions of the ABC transporters, which is essential to better understand the molecular and genetic mechanisms involved in detoxification in *P. xylostella*.

## Methods

### Experimental DBM strains

The experimental population of *P. xylostella* was derived from a susceptible strain (Fuzhou-S) that was collected from a vegetable field of Fuzhou (26.08°N, 119.28°E) in 2004 and used for genome sequencing [27]. Since then this initial population has been reared on potted radish seedlings (*Raphanus sativus* L.) at 25 ± 1 °C, 65 ± 5%RH and L:D = 16:8 h in a separate greenhouse without exposure to insecticides. The two insecticide-resistant strains (CRS and FRS) were selected from this susceptible strain, and detailed in the published *P. xylostella* transcriptome [55].

### Identification of lepidopteran ABC transporters

To identify the ABC transporter genes in the *P. xylostella* genome (http://iae.fafu.edu.cn/DBM/), a systematic BLASTP search was performed using arthropod ABC transporter protein sequences available from NCBI as queries, with the cutoff set at e-value < e^−20^. Candidate ABC transporter sequences were submitted to the NCBI protein database to search for ABC transporter domains. We used the online FGENESH and FGENESH+ programs (http://linux1.softberry.com/berry.phtml) to predict the gene structures of ABC transporter genes on their genomic DNA sequences. Finally, we used program Pfam (http://pfam.xfam.org/) to identify the NBD and TMD structure. The same method was applied to identify ABC transporters from up to date genome versions of *Manduca sexta* (ftp://ftp.bioinformatics.ksu.edu/pub/Manduca/OGS2/), *Danaus plexippus* (http://monarchbase.umassmed.edu/resource.html), and *Heliconius melpomene* (http://www.butterflygenome.org/node/4).

### Sequence alignment and phylogenetic tree construction

To assign the ABC transporter genes to specific subfamilies, the complete aa of NBDs of all *P. xylostella* ABC transporters were aligned by MUSCLE algorithm. Phylogenetic trees were constructed with the Maximum Likelihood method using MEGA-CC (7.0.18) for Linux users. Bootstrap analysis with 1,000 replicates was used to evaluate the significance of the nodes. Poisson correction aa model and all sites were used for the tree reconstruction. Comparison analyses were conducted amon*g P. xylostella*, *B. mori*, *M. sexta*, *H. melpomene*, *D. plexippus*, *D. melanogaster*, *T. urticae* and *H. sapiens* for each of the ABC transporter subfamilies separately using the full-length aa sequences and the same method as what phylogenetic analysis of *P. xylostella* ABC transporters used.

### Examination of gene structure and motifs

The *P. xylostella* ABC genes were mapped onto corresponding scaffolds of the genome sequence assembly version 2 [27]. Gene structure of the *P. xylostella* ABC genes was visualized using the online tool Gene Structure Display Server (http://gsds.cbi.pku.edu.cn/). We used the MEME software (http://meme.sdsc.edu) to identify the conserved motifs in the 82 *PxABC* sequences using the following parameters: number of repetitions = any, maximum number of motifs = 10, and optimum motif width = 3 to 10 residues.

### Gene expression profiling of the *PxABCs*

The RNA-seq data of the *P. xylostella* ABC genes were downloaded from the published database (http://iae.fafu.edu.cn/DBM/). Expressions of the *PxABCs* were profiled in different developmental stages and tissues of the susceptible strain, including eggs (within 24 h after oviposition), 1st ~ 4th-instar larvae, pupae, male and female adults, fat body, hemolymph, Malpighian tubules, silk glands, salivary glands and midgut of the 4th-instar larvae, head of the 4th-instar larvae and male/female adults, and individuals between 3rd-instar larvae of SS, and each of the insecticide-resistant strains (FRS and CRS). Each of the RPKM values was transformed into base-2 logarithm, and the expression profiling of ABC transporter genes was generated and visualized by pheatmap package of the R program (https://www.r-project.org/, version 3.2.5) using the similarity metric of Euclidean distance and clustering method of complete linkage.

### Validation of the gene expression by qRT-PCR

To confirm expression patterns of the *PxABCs* based on *P. xylostella* transcriptome, qRT-PCR analysis was performed using SYBR-green fluorescence with gene-specific primers. The PCR products were examined by dissociation curve analysis after the PCR reaction to confirm the specific detection of target transcripts by the qRT-PCR analysis. Three independent biological replicates were included for qRT-PCR, each of which had three technical replicates. The first-strand cDNA was synthesized from total RNA using the reverse transcriptase kit from Promega (Madison, WI). The qRT-PCR reactions were prepared using the SYBR SELECT MASTER MIX FOR CFX from Invitrogen (Carlsbad, CA) following manufacturer’s instructions and run on a CFX96 Touch™ Real-Time PCR Detection System (Bio-Rad, USA), following the program: 95 °C for 3 min; 45 cycles of 95 °C for 15 s, and 57 °C for 35 s, and a final melt curve at 60 °C for 5 s to 95 °C with 0.5 °C increments. The *P. xylostella* ribosomal protein L32 (RPL32) gene (GenBank acc. no. AB180441) was used as an internal reference. Standard curves were generated by 5-fold dilutions of the cDNA templates. The 2^−ΔCt^ method was used to analyze the relative values of mRNA expression.

## Additional files

Additional file 1: Protein sequences of the 82 ABC transporters of *P. xylostella*. (FA 17 kb)
Additional file 2: Protein sequences of the 19 ABC transporter fragments of *P. xylostella*. (FA 78 kb)
Additional file 3: Phylogenetic tree and gene structure of ABCs in *P. xylostella*. The left side of the figure presents the phylogenetic relationship among ABC transporters in *P. xylostella* and the right, the corresponding gene structures. The green bars represent exons, and black lines represent introns. The digits (0, 1 and 2) on each of the black lines represent three different phases of the introns existed in eukaryotic genes. The length of genes is measured with a scale on the bottom right. Protein sequences used for phylogenetic analysis are provided in the Additional file 1. (FA 30 kb)
Additional file 4: Protein sequences of the ABCA transporters of the eight species. (XLSX 25 kb)
Additional file 5: Protein sequences of the full ABCB transporters of the eight species. (XLSX 13 kb)
Additional file 6: Protein sequences of the half ABCB transporters of the eight species. (DOCX 163 kb)
Additional file 7: Protein sequences of the ABCC transporters of the eight species. (DOCX 273 kb)
Additional file 8: Protein sequences of the ABCD transporters of the eight species. (FA 80 kb)
Additional file 9: Protein sequences of the ABCE transporters of the eight species. (FA 7 kb)
Additional file 10: Protein sequences of the ABCF transporters of the eight species. (DOCX 1284 kb)
Additional file 11: Protein sequences of the ABCG transporters of the eight species. (FA 126 kb)
Additional file 12: Protein sequences of the ABCH transporters of the seven species except *H. sapiens*. (FA 54 kb)
Additional file 13: Log_2_ RPKM values of 82 *PxABCs* at different developmental stages and in different tissues of the susceptible *P. xylostella*. (FA 27 kb)
Additional file 14: Log_2_ RPKM values of 82 *PxABCs* in larvae of the susceptible strain and two insecticide (chlorpyrifos and fipronil)-resistant strains of *P. xylostella*. (FA 172 kb)
Additional file 15: Expression profiles of 18 selected ABC genes at different developmental stages of the susceptible *P. xylostella* as determined by qRT-PCR. X axis: samples; Y axis: relative expression values; E: eggs; 11: the 1st-instar larvae; 21–22: first and second day of the 2nd-instar larvae; 31–32: first and third day of the 3rd-instar larvae; 41–44: each day of the 4th-instar larvae; P1-p4: each day of pupa stage; AM: virgin male adult; AF: virgin female adult; A*M: post-mating male adult; A*F: post-mating female adult. (FA 12 kb)
Additional file 16: Expression profiles of 18 selected ABC genes in the susceptible strain and two insecticide (chlorpyrifos and fipronil)-resistant strains of *P. xylostella* as determined by qRT-PCR. X axis: samples; Y axis: relative expression values; 3-rd: third day of the 3rd-instar larvae; 4-th: third day of the 4th-instar larvae; Pupae: third day of the pupa stage; Males: virgin male adults; Females: virgin female adults; SS: susceptible strain; CRS: chlorpyrifos-resistant strains; FRS: fipronil-resistant strain. (FA 5 kb)

## Abbreviations

20E: 20-hydroxyecdysone
aa: Amino acids
ABC: ATP-binding cassette
AnstABC: ABC transporter of *A. stephensi*
BCRP: Breast cancer resistance protein
BmABC: ABC transporter of *B. mori*
BPU: Benzoylphenylurea
Bt: *Bacillus thuringiensis*
CFTR: Cystic fibrosis transmembrane conductance regulator
CRS: Chlorpyrifos-resistant strains
DDT: Dichlorodiphenyltrichloroethane
FRS: Fipronil-resistant strain
HsABC: ABC transporter of *H. sapiens*
MDR: Multidrug resistance
MRP: Multidrug-resistance associated protein
NBD: Nucleotide-binding domain
PxABC: ABC transporter of *P. xylostella*
PxPgp1: P-glycoprotein 1 of *P. xylostella*
qRT-PCR: Quantitative real-time polymerase chain reaction
RPKM: Reads per kilobase per millionread
SS: Susceptible strain
SUR: Sulfonylurea receptor
TcABC: ABC transporter of *T. castaneum*
TMD: Transmembrane domain
