# Supplementary figures and images for: Characterization and expression profiling of ATP-binding cassette transporter genes in the diamondback moth, Plutella xylostella (L.)
Source: BMC Genomics. 2016 Sep 27;17:760. doi: 10.1186/s12864-016-3096-1 (PMC5039799; doi:10.1186/s12864-016-3096-1)

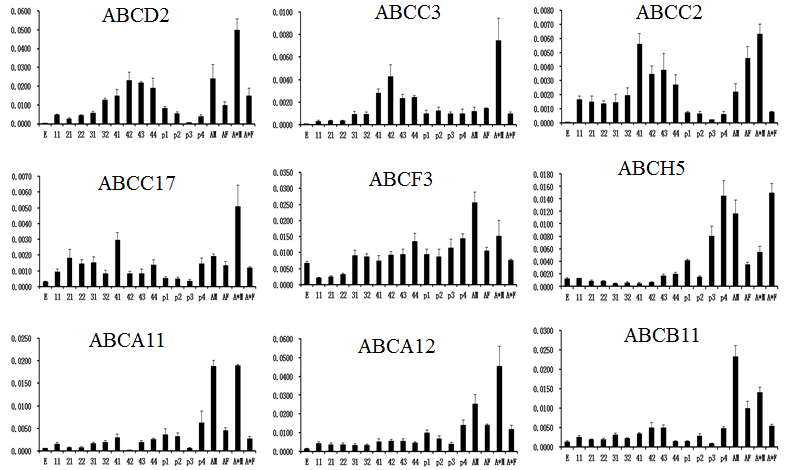


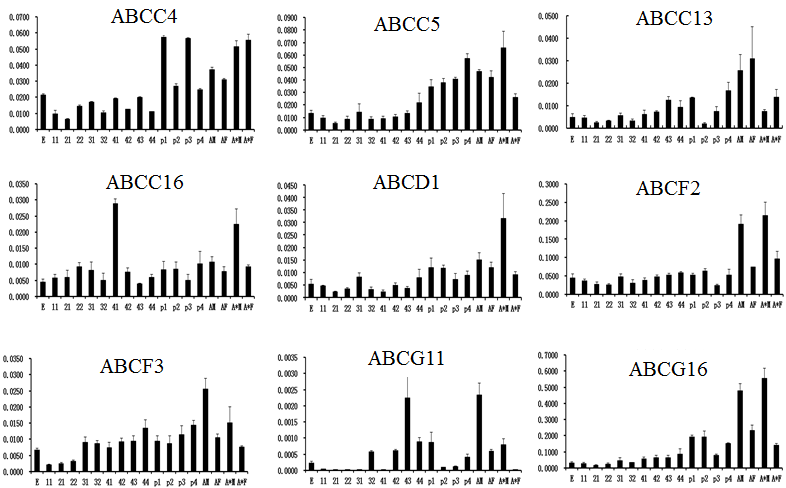

Supplement: Additional file 6: — Protein sequences of the half ABCB transporters of the eight species. (DOCX 163 kb) [file 12864_2016_3096_MOESM15_ESM.docx]

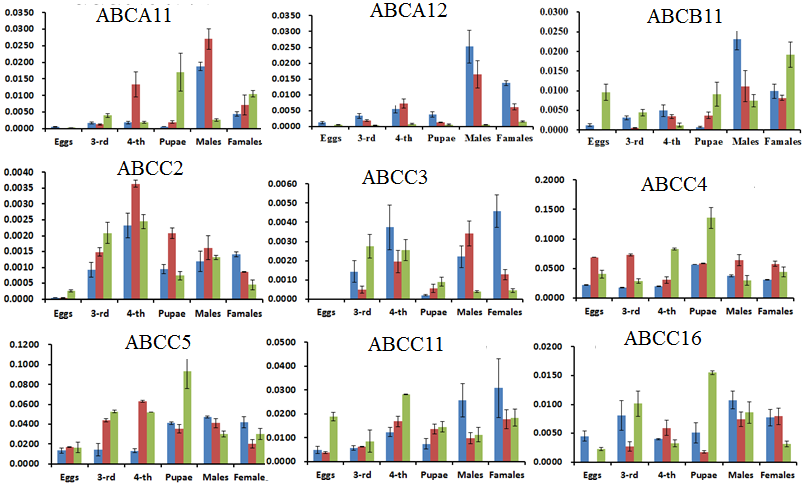


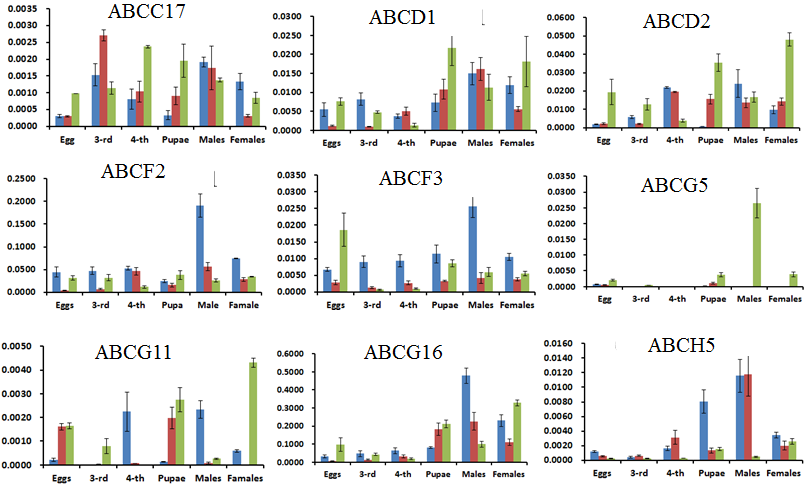


FRS


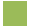


CRS


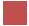


SS


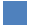

Supplement: Additional file 7: — Protein sequences of the ABCC transporters of the eight species. (DOCX 273 kb) [file 12864_2016_3096_MOESM16_ESM.docx]

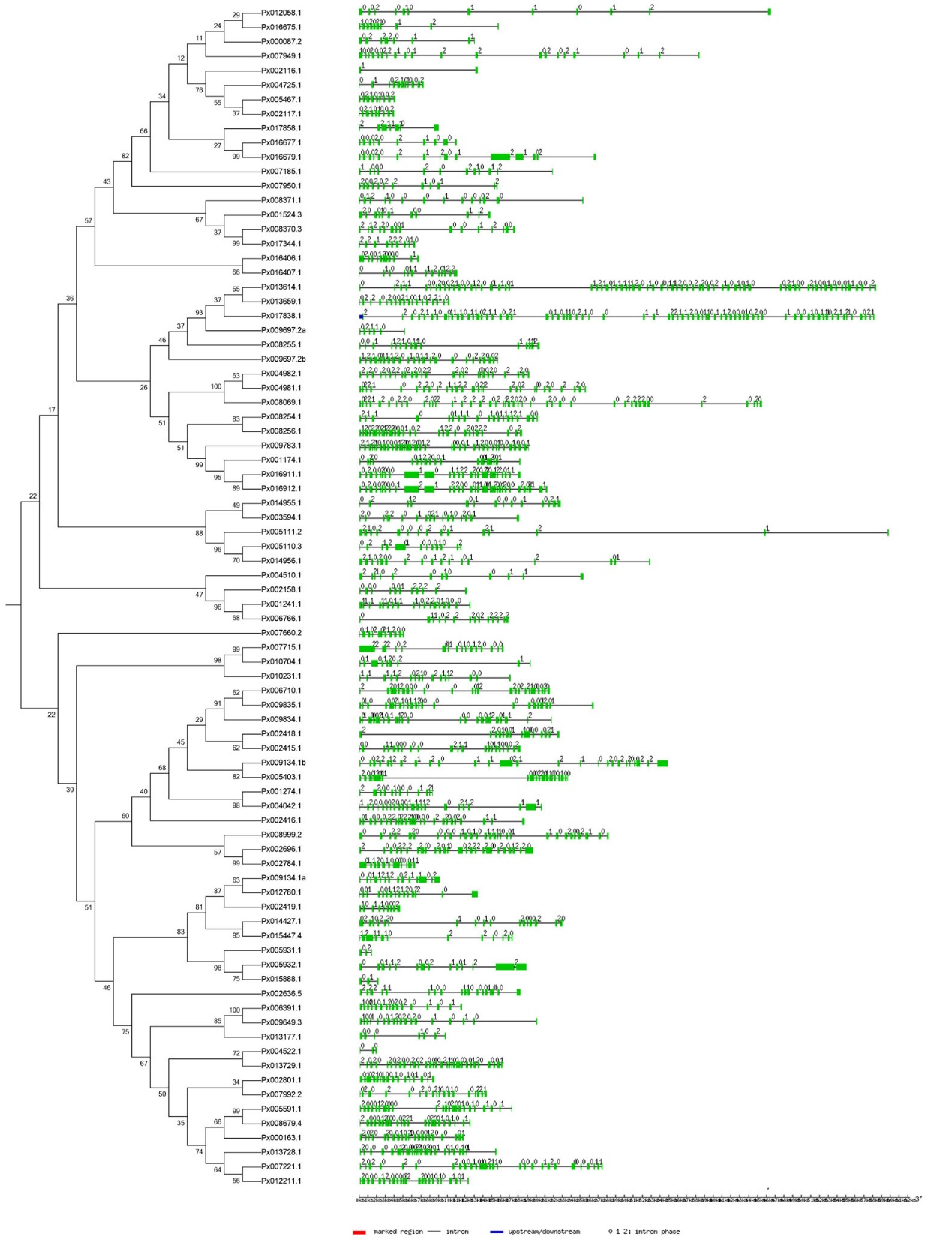


**G**

**A**

**H**

**F**

**E**

**D**

**C**

**B**

0kb 5kb 10kb 15kb 20kb 25kb 30kb 35kb 40kb 45kb 50kb 55kb 65kb

Supplement: Additional file 10: — Protein sequences of the ABCF transporters of the eight species. (DOCX 1284 kb) [file 12864_2016_3096_MOESM3_ESM.docx]
